# Supplementary material for: Workforce outcomes among substance use peer supports and their contextual determinants: A scoping review protocol
Source: PLoS One. 2024 Dec 9;19(12):e0311821. doi: 10.1371/journal.pone.0311821 (PMC11627360; doi:10.1371/journal.pone.0311821)
Supplement: S1 Appendix — (DOCX) [file pone.0311821.s002.docx]

**S2 Appendix. Full search strategies by database.**

| **PsycINFO** |
| --- |
| (SU("peers") OR SU("peer relations") OR SU("peer counseling") OR AB("peer*") OR TI("peer*") OR AB(people N3 lived N3 experience) OR TI(people N3 lived N3 experience) OR AB("personal N3 experience") OR TI("personal N3 experience") OR AB("PWLE") OR TI("PWLE") OR AB("PWLLE") OR TI("PWLLE") ) AND (SU("employee absenteeism") OR SU("employee retention") OR SU("personnel recruitment") OR SU("job satisfaction") OR SU("quality of work life") OR SU("role expectations") OR SU("role perception") OR SU("role satisfaction") OR SU("employee leave benefits") OR SU("emotional adjustment") OR SU("occupational stress") OR SU("employee turnover") OR SU("psychological stress") OR SU("compassion fatigue") OR SU("psychological reactance") OR SU("employee engagement") OR SU("employee well being") OR SU("employee attitudes") OR AB("presenteeism") OR TI("presenteeism") OR AB("absenteeism") OR TI("absenteeism") OR AB("burnout") OR TI("burnout") OR AB("workload") OR TI("workload") OR AB("turnover") OR TI("turnover") OR AB("retention") OR TI("retention") OR AB("recruitment") OR OR TI("recruitment") AB(job N3 satisfaction) OR TI(job N3 satisfaction) OR AB(quality N3 work* N3 life) OR TI(quality N3 work* N3 life) OR AB(secondary N3 traum*) OR TI(secondary N3 traum*) OR AB(intent N3 stay) OR TI(intent N3 stay) OR AB(intent N3 leave) OR TI(intent N3 leave) OR AB(role N3 clarity) OR TI(role N3 clarity) OR AB(role N3 expectation) OR TI(role N3 expectation) OR AB(role N3 perception) OR TI(role N3 perception) OR AB(role N3 satisfaction) OR TI(role N3 satisfaction) OR AB(sick N3 leave*) OR TI(sick N3 leave*) OR AB(collaborative N3 practice) OR TI(collaborative N3 practice) OR AB(staff N3 mix) OR TI(staff N3 mix) OR AB(psychological N3 adaptation*) OR TI(psychological N3 adaptation*) OR AB("psychological adjustment") OR TI("psychological adjustment") OR AB(occupational N3 stress) OR TI(occupational N3 stress) OR OR AB("psychological stress") OR TI("psychological stress") OR AB("industrial psychology") OR TI("industrial psychology") OR AB("occupational psychology") OR TI("occupational psychology") OR AB("compassion fatigue") OR TI("compassion fatigue") OR AB("medical leave") OR TI("medical leave") OR AB(job N3 stress) OR TI(job N3 stress) OR AB(mental N3 stress) OR TI(mental N3 stress) OR AB("psychological resilience") OR TI("psychological resilience") OR AB("psychological reactance") OR TI("psychological reactance") OR AB(work N3 engagement) OR TI(work N3 engagement) OR AB(employee N3 wellbeing) OR TI(employee N3 wellbeing) OR AB(employee N3 attitudes) OR TI(employee N3 attitudes) OR AB(employee N3 engagement) OR TI(employee N3 engagement)) AND (SU("personnel") OR SU("human resource management") OR SU("volunteers") OR AB("workforce") OR TI("workforce") OR AB("personnel") OR TI("personnel") OR AB("human resources") OR TI("human resources") OR AB("volunteer*") OR TI("volunteer*") OR AB("workplace") OR TI("workplace") OR AB("staff") OR TI("staff") OR AB(labor N3 supply) OR TI(labor N3 supply) OR AB(work N1 force) OR TI(work N1 force)) |
| **EMBASE** |
| ((peer NEAR/3 group) OR (peer NEAR/3 counseling) OR (peer NEAR/3 acceptance) OR (peer NEAR/3 pressure) OR (personal NEAR/3 experience) OR 'peer group'/exp OR 'peer counseling'/exp OR 'peer acceptance'/exp OR 'personal experience'/exp OR 'peer pressure'/exp OR 'peer*':ti,ab OR 'people with lived experience':ti,ab OR 'pwle':ti,ab OR 'pwlle':ti,ab) AND ('presenteeism'/exp OR 'absenteeism'/exp OR 'burnout'/exp OR 'workload'/exp OR 'recruitment'/exp OR 'job satisfaction'/exp OR 'quality of working life'/exp OR 'quality of work life'/exp OR 'compassion fatigue'/exp OR 'secondary traumatic stress'/exp OR 'medical leave'/exp OR 'psychological adjustment'/exp OR 'job stress'/exp OR 'mental stress'/exp OR 'psychological resilience'/exp OR 'work engagement'/exp OR 'empowerment'/exp OR 'labor management'/exp OR 'personnel management'/exp OR 'emotional stress'/exp OR 'presenteeism':ti,ab OR 'absenteeism':ti,ab OR 'burnout':ti,ab OR 'workload':ti,ab OR 'recruitment':ti,ab OR 'quality of working life':ti,ab OR 'quality of work life':ti,ab OR 'compassion fatigue':ti,ab OR 'secondary trauma*':ti,ab OR 'medical leave':ti,ab OR 'psychological adjustment':ti,ab OR 'empowerment':ti,ab OR 'turnover':ti,ab OR 'retention':ti,ab OR 'intent* to stay':ti,ab OR 'intent* to leave':ti,ab OR 'sick leave':ti,ab OR 'staff mix':ti,ab OR 'occupational stress':ti,ab OR 'psychological stress':ti,ab OR (job NEAR/3 satisfaction) OR (secondary NEAR/3 trauma) OR (role NEAR/3 (clarity OR expectation OR perception OR satisfaction)) OR (collaborative NEAR/3 practice) OR (staff NEAR/3 mix) OR (psychological NEAR/3 adaptation) OR (psychological NEAR/3 resilience) OR (work NEAR/3 engagement) OR (personnel NEAR/3 management) OR (labor NEAR/3 management) OR (employee NEAR/3 well*)) AND ((human NEAR/3 resources) OR (labor NEAR/3 supply) OR 'workplace'/exp OR 'workforce'/exp OR 'staff'/exp OR 'personnel'/exp OR 'volunteer'/exp OR 'workplace':ti,ab OR 'workforce':ti,ab OR 'staff':ti,ab OR 'personnel':ti,ab OR 'volunteer':ti,ab) |
| **CINAHL** |
| ((MH "peer group+") OR (MH "peer counseling+") OR (MH "peer pressure+") OR (MH "peer assistance programs+") OR (MH "social status+") OR (MH "life experiences+") OR AB("peer*") OR TI("peer*") OR (life N3 experience) OR (social N3 status) OR (Live* N3 experience) OR AB(“PWLE”) OR TI (“PWLE”) OR AB(“PWLLE”) OR TI(“PWLLE”)) AND ((MH "presenteeism+") OR (MH "absenteeism+") OR (MH "workload+") OR (MH "retention+") OR (MH "recruitment+") OR (MH "job satisfaction+") OR (MH "quality of working life+") OR (MH "sick leave+") OR (MH "adaptation, psychological+") OR (MH "stress, occupational+") OR (MH "personnel turnover+") OR (MH "stress, psychological+") OR (MH "personnel retention+") OR (MH "compassion fatigue+") OR (MH "work engagement+") OR (MH "employee attitudes+") OR (MH "role stress+") OR AB("presenteeism") OR TI("presenteeism") OR AB("absenteeism") OR TI("absenteeism") OR AB("workload") OR TI("workload") OR AB("retention") OR  TI("retention") OR AB("recruitment") OR TI("recruitment") OR AB("sick leave") OR TI("sick leave") OR AB("burnout") OR TI("burnout") OR AB("turnover") OR TI("turnover") OR AB("medical leave") OR TI("medical leave") OR AB("sick leave") OR TI("sick leave")OR (employee N3 attitudes) OR (employee N3 well*) OR (emotional N3 stress) OR (work N3 engagement) OR (psychological N3 resilience) OR (mental N3 stress) OR (job N3 stress) OR (psychological N3 adjustment) OR (compassion N3 fatigue) OR (personnel N3 selection) OR (psychological N3 stress) OR (occupational N3 stress) OR (psychological N3 adaptation) OR (staff N3 mix) OR (collaborative N3 practice) OR (role N3 (clarity OR expectation OR perception OR satisfaction OR stress)) OR (intent N3 leave) OR (intent N3 stay) OR (secondary N3 trauma) OR (quality N3 work* N3 life) OR (job N3 satisfaction)) AND ((MH "workforce+") OR (MH "health labor supply+") OR (MH "work environment+") OR (MH "health personnel, unlicensed+") OR (MH "employees+") OR AB("workplace") OR TI("workplace") OR AB("workforce") OR TI("workforce") OR AB("staff") OR TI("staff") OR AB("personnel") OR TI("personnel") OR AB("employee*") OR TI("employee*") OR AB("volunteer*") OR TI("volunteer*") OR AB("human resource*") OR TI("human resource*") OR (labor N3 supply)) |
| **Web of Science** |
| (TS=(peer*) OR TS=(lived NEAR/3 experience) OR TS=("PWLE") OR TS=("PWLLE") OR TS=(personal  NEAR/3 experience) ) AND (TS=(presenteeism) OR TS=(absenteeism) OR TS=(burnout) OR TS=(workload)  OR TS=(turnover) OR TS=(retention) OR TS=(recruitment) OR TS=(job NEAR/3 satisfaction) OR TS=(Job  NEAR/3 stress) OR TS=(occupational NEAR/3 stress) OR TS=(personnel NEAR/3 selection) OR TS=(quality  NEAR/3 work*) OR TS=("sick leave") OR TS=("medical leave") OR TS=(psychological NEAR/3 stress) OR  TS=("compassion fatigue") OR TS=(emotional NEAR/3 stress) OR TS=(mental NEAR/3 stress) OR  TS=(employee NEAR/3 well*) OR TS=(Employee NEAR/3 attitude*) OR TS=(role NEAR/3 (expectation OR  perception OR satisfaction)) OR TS=(counseling) OR TS=(intent NEAR/3 stay) OR TS=(Intent NEAR/3  leave) OR TS=(psychological NEAR/3 adaptation) OR TS=(Psychological NEAR/3 resilience) OR TS=("staff  mix") OR TS=(collaborative NEAR/3 practice) OR TS=(secondary NEAR/3 trauma)) AND (TS=(workplace)  OR TS=(workforce) OR TS=(staff) OR TS=(personnel) OR TS=(labor NEAR/3 supply) OR TS=(human  NEAR/3 resource*) OR TS=(volunteer)) |
| **Google Scholar** |
| (peer\|"lived experience") AND (present\|absent\|burnout\|turnover\|retention\|stress\|sick\|counsel\|resilience) AND (workplace\|workforce\|staff\|personnel\|human resource\|volunteer) |
